# A computational study of hedgehog signalling involved in basal cell carcinoma reveals the potential and limitation of combination therapy

**DOI:** 10.1186/s12885-018-4451-1

**Published:** 2018-05-18

**Authors:** Antoine Buetti-Dinh, Rebecca Jensen, Ran Friedman

**Affiliations:** 1 Department of Chemistry and Biomedical Sciences, Linnæus University, Norra vägen 49, Kalmar, SE-391 82 Sweden; 2 Linnæus University Centre for Biomaterials Chemistry, Linnæus University, Norra vägen 49, Kalmar, SE-391 82 Sweden; 3 Centre for Ecology and Evolution in Microbial Model Systems, Linnæus University, Landgången 3, Kalmar, SE-391 82 Sweden; 4 Institute of Computational Science, Faculty of Informatics, Università della Svizzera Italiana, Via Giuseppe Buffi 13, Lugano, CH-6900 Switzerland; 5 Swiss Institute of Bioinformatics, Quartier Sorge – Batiment Genopode, Lausanne, CH-1015 Switzerland

**Keywords:** Basal cell carcinoma, Drug resistance, Knowledge-based analysis, Vismodegib, Sonidegib, Erivedge, Odozmo

## Abstract

**Background:**

The smoothened (SMO) receptor is an essential component of the Sonic hedgehog (SHH) signalling, which is associated with the development of skin basal cell carcinoma (BCC). SMO inhibitors are indicated for BCC patients when surgical treatment or radiation therapy are not possible. Unfortunately, SMO inhibitors are not always well tolerated due to severe side effects, and their therapeutical success is limited by resistance mutations.

**Methods:**

We investigated how common are resistance-causing mutations in two genomic databases which are not linked to BCC or other cancers, namely 1000 Genomes and ExAC. To examine the potential for combination therapy or other treatments, we further performed knowledge-based simulations of SHH signalling, in the presence or absence of SMO and PI3K/Akt inhibitors.

**Results:**

The database analysis revealed that of 18 known mutations associated with Vismodegib-resistance, three were identified in the databases. Treatment of individuals carrying such mutations is thus liable to fail a priori. Analysis of the simulations suggested that a combined inhibition of SMO and the PI3K/Akt signalling pathway may provide an effective reduction in tumour proliferation. However, the inhibition dosage of SMO and PI3K/Akt depended on the activity of phosphodiesterases (PDEs). Under high PDEs activities, SMO became the most important control node of the network. By applying PDEs inhibition, the control potential of SMO decreased and PI3K appeared as a significant factor in controlling tumour proliferation.

**Conclusions:**

Our systems biology approach employs knowledge-based computer simulations to help interpret the large amount of data available in public databases, and provides application-oriented solutions for improved cancer resistance treatments.

**Electronic supplementary material:**

The online version of this article (10.1186/s12885-018-4451-1) contains supplementary material, which is available to authorized users.

## Background

Smoothened (SMO), a 7-pass transmembrane protein, is a component of the Hedgehog (HH) and Patched (PTCH) signalling network responsible for the regulation of cell growth and embryonic development. Mutations that affect the members of this signalling network are associated with the development of skin basal cell carcinomas (BCC) [1] through Gli transcription factors that drive an uncontrolled cell proliferation.

Amino acid mutations in SMO that lead to an unrestrained receptor activity are called activating mutations [2]. Although inhibitors such as vismodegib [3] (Erivedge ^*®*;^, FDA-approved in 2012) and sonidegib [4] (Odozmo ^*®*;^, FDA-approved in 2015) that block SMO are available [5], resistance mutations develop after a period of few months thereby limiting the efficacy of those drugs [6]. In addition to amino acid mutations in the receptor, resistance can arise also by a second mechanism that relies on alternative signalling pathways. Biological signalling is typically distributed over multiple components involving converging, diverging and recursive branches of the signalling network. Under selective pressure, tumours can reinforce an alternative signalling route as a response to the blockage of the main signal transduction path. This mechanism of resistance allows cancers to sustain cancer-driving processes such as tumour proliferation despite therapy [7, 8].

Resistance mutations provide a selective advantage to the tumour in the face of treatment [9], but may be somewhat deleterious otherwise [10]. To examine whether SMO resistance mutation may pre-exist treatment, or even BCC, the 1000 Genomes [11] and ExAC (Exome Aggregation Consortium) [12] databases were surveyed for such mutations. Owing to the resistance mutations, it is necessary to find therapeutic strategies against BCC that not only rely on inhibiting SMO, but also focus on other targets in the SMO signalling network. We had previously developed a computational knowledge-based framework to study signalling pathways, especially in the context of cancer [13] and used it to study combination therapies [14, 15]. Here, we used this approach to predict which signalling pathways need to be targeted by combination therapy, in order to sufficiently impair the development of BCC even if SMO resistance mutations arise.

## Results and discussion

### Population occurrence of mutations in the SMO domain make it a difficult target for therapeutic inhibition

40 mutations in 35 amino acid positions were identified in SMO according to the COSMIC database [16] (surveyed in February 2017). Some of these mutations are associated with resistance to SMO inhibitors such as vismodegib (Table 1). This explains the limited success of these inhibitors.

**Table 1:** Known resistance mutations in the SMO domain. Source: COSMIC http://cancer.sanger.ac.uk/cosmic[16]

Mutation	Number	Associated with
	of samples	BCC initiation
H231R	2	Yes
T241M	1	No
W281C	2	Yes
W281L	1	No
V321A	1	Yes
V321M	3	Yes
A459V	3	Yes
F460L	1	Yes
C469Y	1	No
D473G	7	Yes
D473H	2	Yes
D473N	1	Yes
D473Y	1	No
Q477E	1	Yes
G497W	3	Yes
S533N	1	Yes
W535L	10	Yes
W535R	1	Yes

We further searched for the presence of resistance mutations in two genomic databases that are not linked to BCC or any other cancer, namely 1000 Genomes and ExAC. This analysis (carried out on February 2017), revealed that three resistance mutations were found in the population: W281C (with calculated frequency *p*=8.24·10^−6^), D473N (*p*=4.13·10^−5^) and D473H (*p*=0.021, which may be an overestimation since the sample size was only 48). This indicated that normal genetic variation was enough to confer resistance to SMO inhibitors. Of note, in the vast majority of cases, resistance mutations lead to a decrease in the drug’s affinity towards its molecular target, which cannot be offset by a higher dose of the drug due to toxicity and intolerance. Combination therapy therefore represents a potential solution to treatment-resistant cancers. Simulation and sensitivity analysis of the SMO signalling network were used to identify optimal signalling targets for a combination therapy against drug-resistant BCC.

### Signalling pathway analysis identified PI3K/Akt and PDEs as potential targets for treatment of BCC

**Fig. 1:**
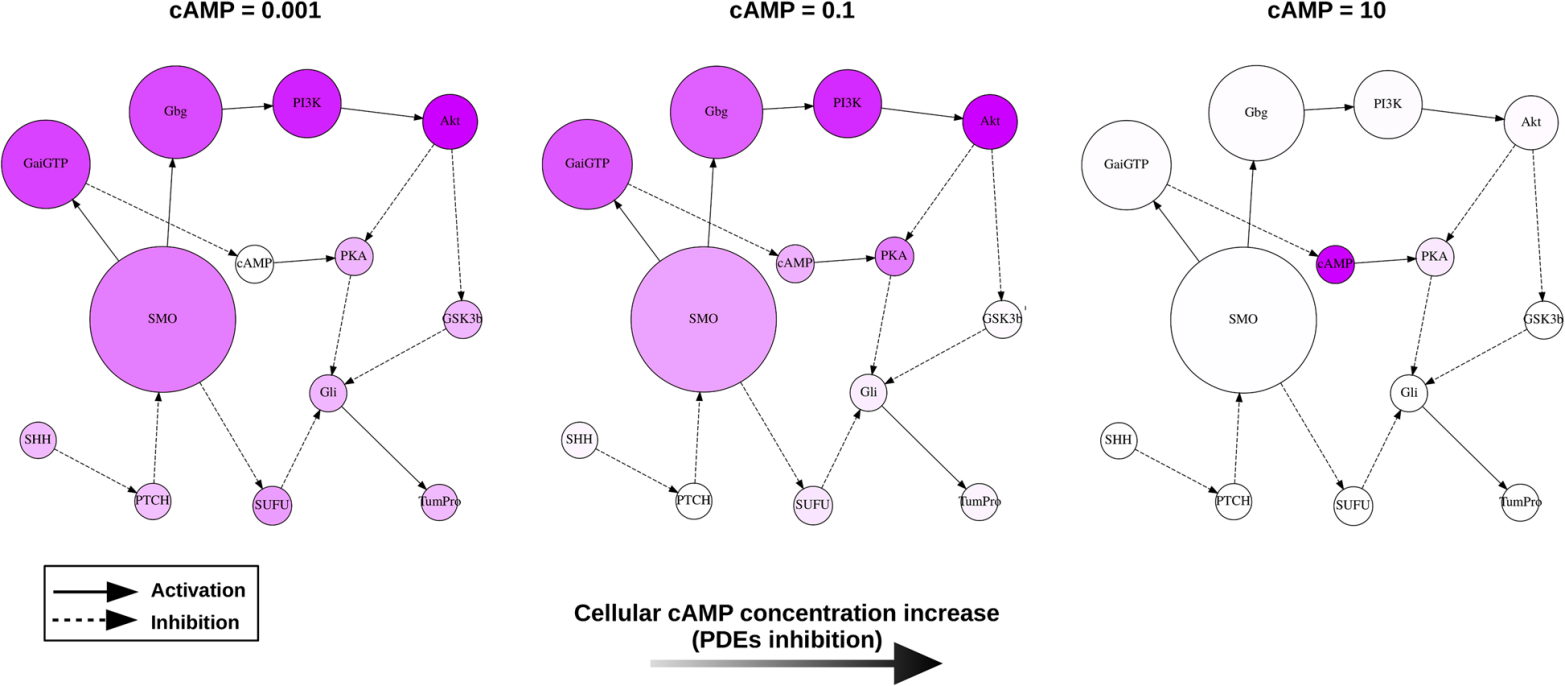
The interaction network of SMO and signalling pathway analysis. Signalling pathway analysis represents the involvement of the network components in stimulating the tumour proliferation end-point (TumPro) upon an increase in SMO independent activity (*β*(*SMO*)=0.001→0.1). See the Methods section for a description of the components. The effect of PDEs inhibition is represented as an increase in the concentration of cAMP (from left to right). The sizes and colour intensities of the nodes summarise the results of signalling pathway analysis and represent how the signal is mediated from SMO to the network end-point. The node size represents the association of a node with SMO when stimulating the end-point (the larger the node the stronger the association). The node colour indicates the signal flow (the darker the more a node can deliver a signal downstream to it)

The SMO signalling network was simulated in order to identify potential molecular targets for therapy (Fig. 1). In the simulations of the signal transduction network, the signals are transmitted between the different components of the network through activation or inhibition, which culminate in the cancer-promoting end-point “tumour proliferation” (TumPro). The simulations were performed by applying a coarse-grained approach [15] whereby exhaustive simulations of the network states were carried out in which each node assumed one of two possible states: “low activity” or “high activity” (see Methods section). This enabled us to identify the network states that were most significant for the development of high tumour proliferation, and consequently, the signalling pathways relevant for therapeutic intervention.

#### Knowledge-based simulations highlight the important role of PI3K/Akt signalling

Analysis of the simulations suggested that upon an independent activity increase of SMO (for example due to an activating mutation), signalling to the tumour proliferation end-point is mediated principally by the pathway involving the PI3K and Akt kinases (left panel in Fig. 1). The degree of association between each node and SMO’s activity is indicated by its size in the figure. In addition, each node’s signal flow intensity was represented proportionally to the node’s colour intensity, i.e., the darker the node the more active it was when the control node (SMO) was highly active. Both measures indicated the PI3K/Akt pathway as the main signalling route to stimulate tumour proliferation. Thus, analysis of the simulations revealed that a combined inhibition of SMO and of the PI3K/Akt pathway branch would be an effective therapeutic strategy against BCC, in agreement with other studies [5, 17]. Unfortunately, inhibition of the PI3K/Akt pathway is difficult to achieve [18, 19]. Interestingly, the simulations also indicated Cyclic adenosine monophosphate (cAMP) as a potential network control point for reducing tumour proliferation. Cyclic nucleotide phosphodiesterases (PDEs) are enzymes that catalyse the degradation of a phosphodiesther bond in cAMP or cGMP, and are thus potential molecular targets for intervention (inhibition of PDEs leads to an increase of the concentration of free cAMP).

#### Inhibition of PDEs may reduce tumour proliferation

Several, selective and non-selective PDEs inhibitors are currently available and have the effect to increase intracellular cAMP [20, 21]. PDEs inhibitors have been employed to treat several diseases [22] including different types of cancers such as oral squamous carcinoma [23], colon [24, 25] and breast cancer [26]. Roflumilast (Daliresp ^*®*;^, FDA-approved in 2011 to treat chronic obstructive pulmonary diseases) is a PDE4 inhibitor also used to treat B-cell malignancies [27].

By simulating an increase in cAMP (*β*(*cAMP*) from 0.001 to 0.1), the SMO network became less branched: the nodes GSK3b and SUFU became less involved in conveying signalling from SMO to the tumour proliferation end-point (TumPro). In addition, the steady-state activity of the tumour proliferation end-point was reduced (compare the left with the central panel in Fig. 1). Ultimately, simulations of very strong PDEs inhibition, where the resulting cAMP concentration compares to a 100-fold higher activity with respect to the high nodes’ activity level (*β*=0.1) assumed in the network simulation, revealed a bottleneck where the only relevant control node for the signal flow in this extreme situation is cAMP (see right panel in Fig. 1).

### Network control analysis reveals changes in the significance of SMO and PI3K as a function of circulating cAMP

**Fig. 2:**
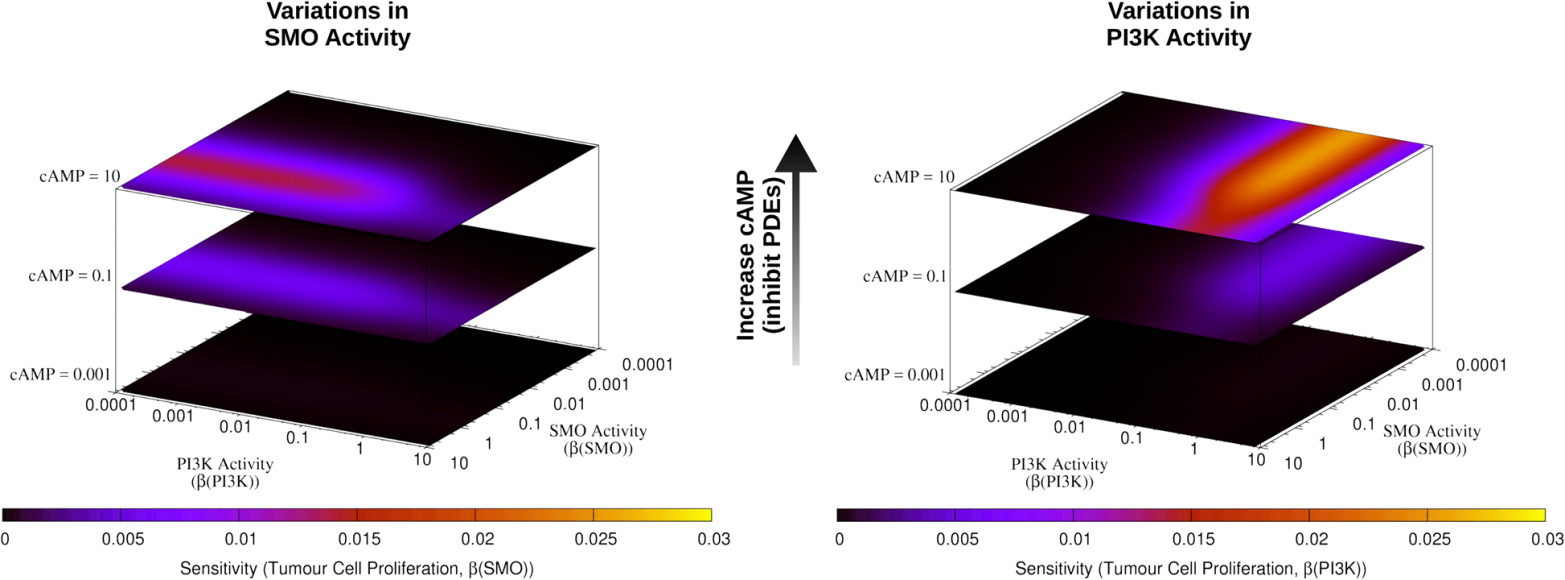
Sensitivity heat maps of the SMO network. Sensitivity was calculated in response to variations in SMO and PI3K activities on the xy-plane (left and right, respectively), combined with different levels of cAMP (as in Fig. 1) on the z-axis. The regions of high sensitivity between low and high SMO and PI3K activities (blue-red-yellow areas) correspond to a transition zone between low and high steady-state activity of tumour proliferation

Sensitivity analysis of the SMO network was carried out by simulating small variations in the activity of the SMO and PI3K. By following earlier studies [13, 14], this enabled us to identify activity ranges where control of tumour proliferation is possible through inhibition of SMO and PI3K, in combination with different levels of cAMP (corresponding to different PDEs inhibition strengths, see Fig. 2).

At low cAMP (no PDEs inhibition), tumour proliferation had a high steady-state activity (not shown), and was rather insensitive to variation in PI3K and SMO (see lower heatmaps in Fig. 2). Increasing cAMP (i.e., applying PDE inhibition), improved the controllability of the system by both SMO and PI3K: a region of high sensitivity formed between low and high SMO and PI3K activities (see left and right panels of Fig. 2, respectively). This corresponds to a transition zone between low and high steady-state activity of tumour proliferation, and indicates the presence of controllable regions (with respect to variations in SMO or PI3K). Combined inhibition that targets SMO and PI3K in correspondence to these regions (with respect to a maximal level of SMO and PI3K activities) is thus suggested to be effective in reducing tumour proliferation.

Network control analysis suggested that the effect of dual inhibition of SMO and PI3K differed depending on the PDEs activity (see peak (maximal) sensitivity values in Table 2 corresponding to the different conditions of Fig. 2). At low and intermediate cAMP (*β*(*cAMP*)=0.001 and *β*(*cAMP*)=0.1, respectively), the system’s highest sensitivity values were obtained by variations in SMO activity, i.e., the network end-point’s controllability primarily depended on variations in SMO activity (0.00024 and 0.00619, respectively); and secondarily on PI3K activity variations (0.00019 and 0.00521, respectively). In contrast, when the cAMP level was high (corresponding to a very strong PDEs inhibition), the end-point’s controllability showed a stronger dependence on PI3K than on SMO activity variations (0.02538 *versus* 0.01380). Therefore, under no and moderate PDEs inhibition (*β*(*cAMP*)≤0.1), SMO was ∼20 more influential than PI3K for controlling tumour proliferation, while at high PDEs inhibition (*β*(*cAMP*)=10) PI3K was ∼20% more determinant than SMO for the end-point control.

**Table 2:** Peak sensitivity values for the conditions represented in Fig. 2

	*β*(*cAMP*)=0.001	*β*(*cAMP*)=0.1	*β*(*cAMP*)=10
Variations in SMO Activity	0.00024	0.00619	0.01380
Variations in PI3K Activity	0.00019	0.00521	0.02538

### Combined inhibition of network control points revealed by principal component analysis

Principal component analysis (PCA) was used to detect co-activity and co-regulatory patterns (when it was applied to steady-state and sensitivity values, respectively) between the signalling components at the different cAMP levels applied in the network control analysis dataset presented in section “Signalling pathway analysis identified PI3K/Akt and PDEs as potential targets for treatment of BCC”. Sensitivity PCA showed that by increasing cAMP from a low level, a cluster formed that included cAMP and PKA (see Additional file 1: Figures S1–S3). This analysis thus supports the earlier conclusion that cAMP influences the signal transduction through PKA and that it can act as a regulator in addition to the PI3K/Akt pathway, as suggested before by signalling pathway analysis in section “Inhibition of PDEs may reduce tumour proliferation”. Furthermore, SMO clustered with nodes directly downstream of it (Gbg and GaiGTP) at low and intermediate cAMP (see Additional file 1: Figures S1 and S2). When cAMP was high, this cluster merged with PI3K and Akt, suggesting that strong PDEs inhibition and consequent increase of cAMP causes the network components PI3K, Akt and SMO and their downstream partners (Gbg and GaiGTP) to become co-regulated (see Additional file 1: Figure S3). A combination of PDEs inhibition that causes an increase in cAMP, together with inhibition of the SMO and the PI3K/Akt pathway is therefore predicted to counter tumour proliferation.

## Conclusions

SMO is subject to various amino acid mutations or variations which apparently do not hamper its activity. Some of these mutations provide a basis for developing resistance mutations against therapeutic inhibitors, which is one reason that makes SMO a challenging target for BCC therapies. We performed simulations of the SHH/SMO interaction network in BCC in order to identify signalling pathways that could confer resistance to treatments. Our approach enabled the identification of potential treatment combinations effective against forms of BCC harbouring resistance mutations in SMO.

The simulations suggested that upon an increase in SMO activity, the PI3K/Akt signalling plays a crucial role in promoting tumour proliferation. Combined inhibition of SMO and PI3K was studied in detail together with the effect of different levels of cAMP (PDE inhibition). Our results indicate a complex regulation of SMO and PI3K as a function of PDEs activity. SMO was the most important control node of the network at no and moderate inhibition of PDEs. However, under strong PDEs inhibition, the systems became more sensitive to both SMO and PI3K, but PI3K became more relevant than SMO for controlling tumour proliferation. PI3K was previously suggested to be involved in acquired resistance to treatments against refractory tumours [5, 17]. Our simulations propose a mechanism based on activation/inhibition of signalling components in the SMO network, where PDEs determine the outcome of therapies that target the PI3K signalling. These results provide quantitative insights into the signalling network of SHH/SMO involved in BCC, and suggest that a combined inhibition with different dosages of SMO, PDE and PI3K/Akt inhibitors may be required to tackle drug-resistant BCC.

## Methods

### Simulations of the SMO signalling network

A signalling network model of SMO and its principal interaction partners described in the literature was constructed based on the current state of knowledge [5, 28–36] (see Fig. 1). SMO is negatively regulated by the transmembrane receptor PTCH. SHH derepresses the PTCH-mediated effect on SMO. Through different intermediates, this leads to the activation of the Gli family of transcription factors, which in turn activate genes involved in tumour proliferation [30, 31]. Downstream of SMO, G *α*_*i*_ proteins decrease the level of cAMP upon Guanosine-5’-triphosphate (GTP) hydrolysis (indicated as GaiGTP); and consequently prevent the inhibitory phosphorylation of Gli by protein kinase A (PKA). In parallel, G *βγ* (Gbg) subunits inhibit PKA through the PI3K/Akt (phosphoinositide 3 kinase / protein kinase B) pathway [32, 33]. This relieves the glycogen synthetase kinase 3 *β* (GSK3b)-mediated inhibition of Gli proteins [34, 35]. Furthermore, SMO also relieves the inhibition of the suppressor of fused (SUFU) onto Gli proteins further enhancing the tumour proliferation effect [30, 36].

The interaction network model of SMO signalling was simulated with the computational method developed by us previously [13, 14]. The network’s nodes represented signalling components as a set of ordinary differential equations (ODEs). Edges represented the interaction links between the components (modelled as empirical Hill-type transfer functions). This enabled the integration of experimental information in the modelling framework in a straightforward way using a well-established formalism derived from classical enzyme kinetics. This approach requires only the knowledge necessary to set up Boolean models (where interaction is assumed to be binary, i.e., activation or inhibition). Despite its simplicity, the analysis of such simulations provides quantitative insights on studied signalling networks, taking into account nonlinear signalling effects such as feedbacks, pleiotropy and redundancy. This way, our method allows to analyse computationally disease networks for which detailed experimental information is not available.

The simulation procedure yielded steady-state activity levels of the different network components according to a given set of parameters. The range of independent activities of the different network components (*β*) is summarised in Additional file 1: Table S1 (see reference [13] for a complete list of parameters available in our code). Sensitivity analysis was applied to the resulting steady-state activities by calculating the sensitivity corresponding to each parameter change. Steady-state simulations and sensitivity analysis were carried out using parallel computational architectures in order to screen a large number of conditions and identify key control points of the network. This enabled us to methodically characterise the effect of inhibition of SMO and PI3K under the different levels of PDEs activity.

### Simulation and sensitivity in signalling pathway analysis

The analysis of signalling pathways involved in tumour proliferation consisted of enumerating all combinations of network states with *high* (*β*=0.1) or *low* (*β*=0.001) initial activity state (see Additional file 1: Table S1). For each pairwise combination of parameters (where the network state differs by the activity of a single node), sensitivity was calculated according to the method used in reference [15], i.e.,

1 $$ {{\varepsilon}}^{SS(N_{i})_{\beta(N_{j})=low} \: \rightarrow \: SS(N_{i})_{\beta(N_{j})=high} }_{{ \beta(N_{j})=low} \: \rightarrow \: \beta(N_{j})=high } = \frac{ ln \left\{ \frac{SS(N_{i})_{\beta(N_{j})=high} }{ SS(N_{i})_{\beta(N_{j})=low}} \right\}}{ ln \left\{ \frac{{\beta(N_{j})=high} }{{\beta(N_{j})=low}} \right\}}  $$

where *SS*(*N*) denotes the steady-state activity of a node *N* and *β*(*N*) its independent activity state. The arrow (→) indicates a change in condition.

Without considering the combined activity change of multiple control nodes simultaneously, but only the changes occurring subsequently one after another (as it would be expected by point mutations affecting the activity of a protein), Eq. 1 allows to calculate the *s*^*n*^ conditions that represent all possible states of the network (*s* is the number of states a node can assume, *n* is the number of nodes in the network).

Sensitivity is subsequently computed for each pair of simulated conditions that differ by a single parameter (i.e., pair of simulations where the network states are identical except for a single node that is low in the first simulation and high in the second, or *vice versa*). This resulted in a set of calculated sensitivities derived from the coarse-grained simulations that comprises $s^{n} \cdot \frac {n}{s} \cdot (s-1)$ sensitivity values from which signal flow graphs are computed (see Fig. 1).

The obtained sensitivity values represent the strength of the influence exerted by a node, connected directly or through intermediates, onto another component of the network. A positive value for the sensitivity between two nodes (A → B) indicates that upon the increase of the activity of A, B’s activity will also increase. Similarly, a negative sensitivity indicates that upon an increase of A’s activity, B’s activity will decrease. Sensitivity values close to 0 indicated independence between nodes. Signal flow graphs (see Fig. 1) were built based on the node’s activity and on the calculated sensitivity values. They represent how the signal travels from the control node (node subject to an increase in independent activity) to the network end-points. Upon activation of the control node, the statistical association of other nodes that are influenced is represented by the graph’s node area (the larger the stronger the association). The colour of the nodes indicates their activity contribution (the darker is a node, the stronger is the signal it can deliver downstream to it).

### Simulation and sensitivity in network control analysis

Based on the same mathematical principles as for in the signalling pathway analysis, in network control analysis the majority of the network components were assumed to have a low (resting) activity, while few nodes, identified by signalling pathway analysis as relevant for controlling the network behaviour, were varied over a range of activities (*β*) in small steps (as explained in reference [15] and expanded in Additional file 1: Table S1). This yielded a more detailed characterisation of those nodes that were critical for controlling the network end-points and consequently relevant for cancer development.

### Principal component analysis and hierarchical clustering

PCA was used as a multivariate analysis to reduce dimensionality of the simulation dataset of the network control analysis (the *prcomp* function of R was used as a part of the computational method developed by us previously [13, 14]). It was applied to visualise PCA loadings (corresponding to the network components) of steady-state and sensitivity data on a two-component space (as presented in the top panels in Additional file 1: Figures S1–S3). PCA loadings were further classified using hierarchical clustering (the *hclust* function of R was used) and represented in a tree-like structure (dendrogram) whose branches grouped network components according to their similarity over the different simulations (displayed in the bottom dendrograms of Additional file 1: Figures S1–S3).

## Additional file

Additional file 1 Supplementary Material. PCA at different levels of cAMP and model parameters. (PDF 1380 kb)

## Abbreviations

BCC: Basal cell carcinoma
cAMP: Cyclic adenosine monophosphate
ExAC: Exome Aggregation Consortium
GTP: Guanosine-5’-triphosphate
HH: Hedgehog
ODEs: Ordinary differential equations PTCH: Patched
PDEs: Cyclic nucleotide phosphodiesterases
PCA: Principal component analysis
PKA: Protein kinase A
SMO: Smoothened
SUFU: Suppressor of fused
