# Supplementary material for: A computational study of hedgehog signalling involved in basal cell carcinoma reveals the potential and limitation of combination therapy
Source: BMC Cancer. 2018 May 18;18:569. doi: 10.1186/s12885-018-4451-1 (PMC5960207; doi:10.1186/s12885-018-4451-1)
Supplement: Supplementary file 1 — Supplementary Material. PCA at different levels of cAMP and model parameters. (PDF 1380 kb) [file 12885_2018_4451_MOESM1_ESM.pdf]

A Computational Study of Hedgehog Signalling Involved in Basal Cell Carcinoma Reveals the Potential and Limitation of Combination Therapy

Antoine Buetti-Dinh [1,2,3,4,5], Rebecca Jensen [1], and Ran Friedman [1,2]

[1]Department of Chemistry and Biomedical Sciences, Linnæus University, Kalmar, Sweden antoine.buetti@lnu.se
[2]Linnæus University Centre for Biomaterials Chemistry, Linnæus University, Kalmar, Sweden ran.friedman@lnu.se
[3]Centre for Ecology and Evolution in Microbial Model Systems, Linnaeus University, Sweden
[4]Institute of Computational Science, Faculty of Informatics, Università della Svizzera Italiana, Lugano, Switzerland
[5]Swiss Institute of Bioinformatics, Lausanne, Switzerland

SUPPLEMENTARY MATERIAL

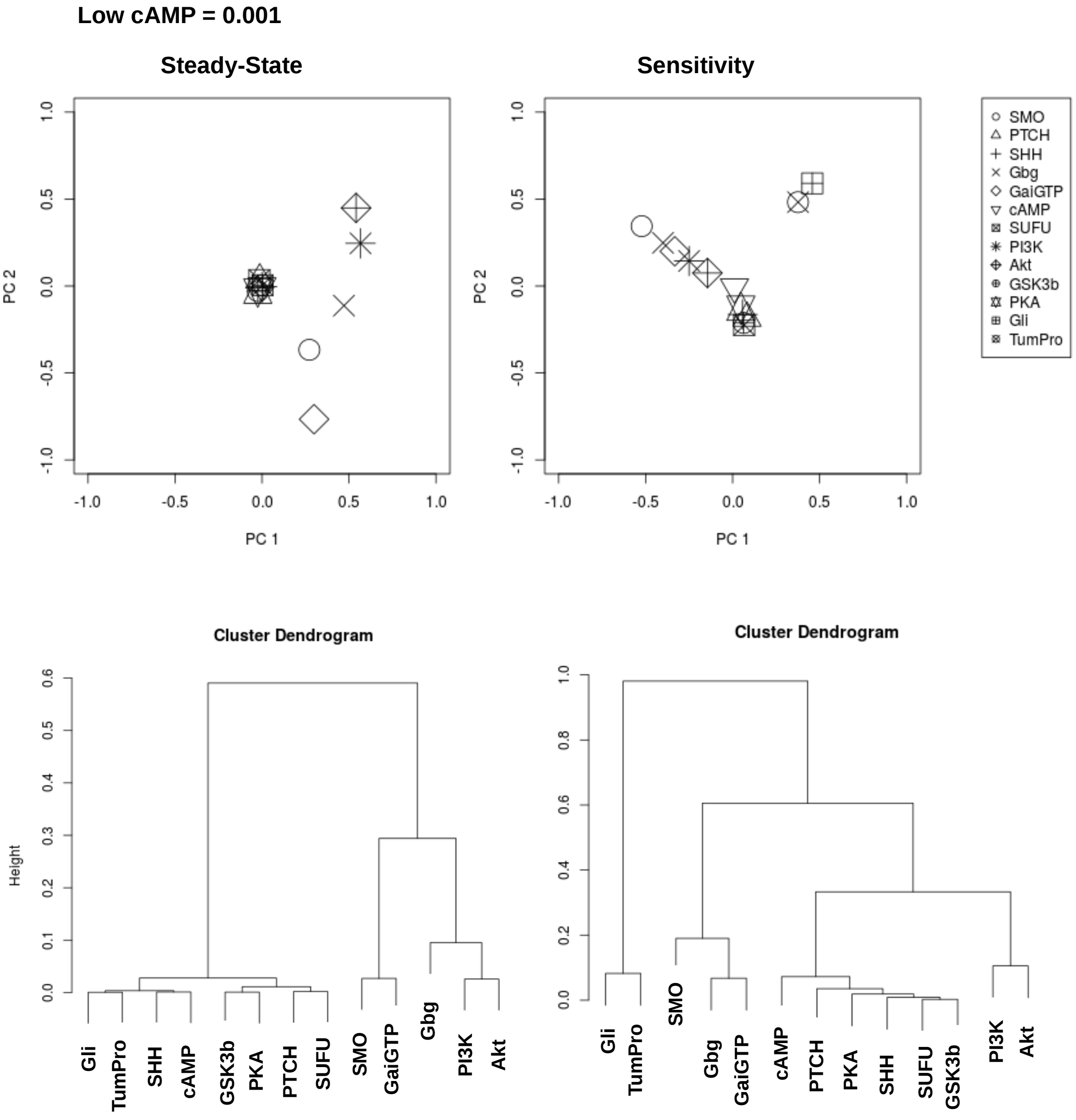

Supplementary Figure S1: **PCA at low levels of cAMP (no PDEs inhibition).** Steady-state (left) and sensitivity (right) PCA of the network control analysis dataset presented in section "Signalling pathway analysis identified PI3K/Akt and PDEs as potential targets for treatment of BCC" at low cAMP. Hierarchical clustering dendrograms (bottom panels) on the PCA loadings (upper panels).

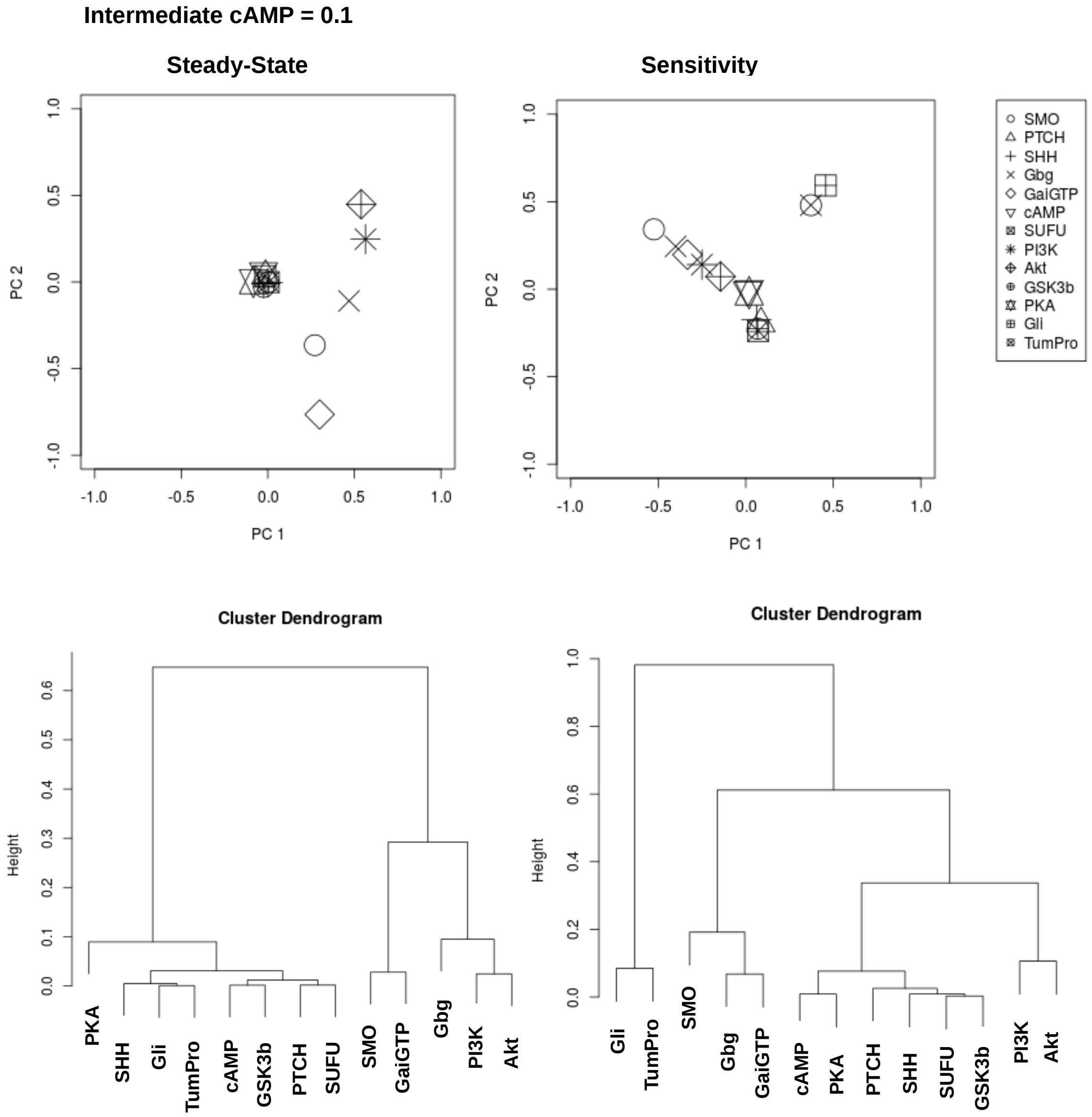

Supplementary Figure S2: **PCA at intermediate levels of cAMP (mild PDEs inhibition).** Steady-state (left) and sensitivity (right) PCA of the network control analysis dataset presented in section "Signalling pathway analysis identified PI3K/Akt and PDEs as potential targets for treatment of BCC" at intermediate cAMP. Hierarchical clustering dendrograms (bottom panels) on the PCA loadings (upper panels).

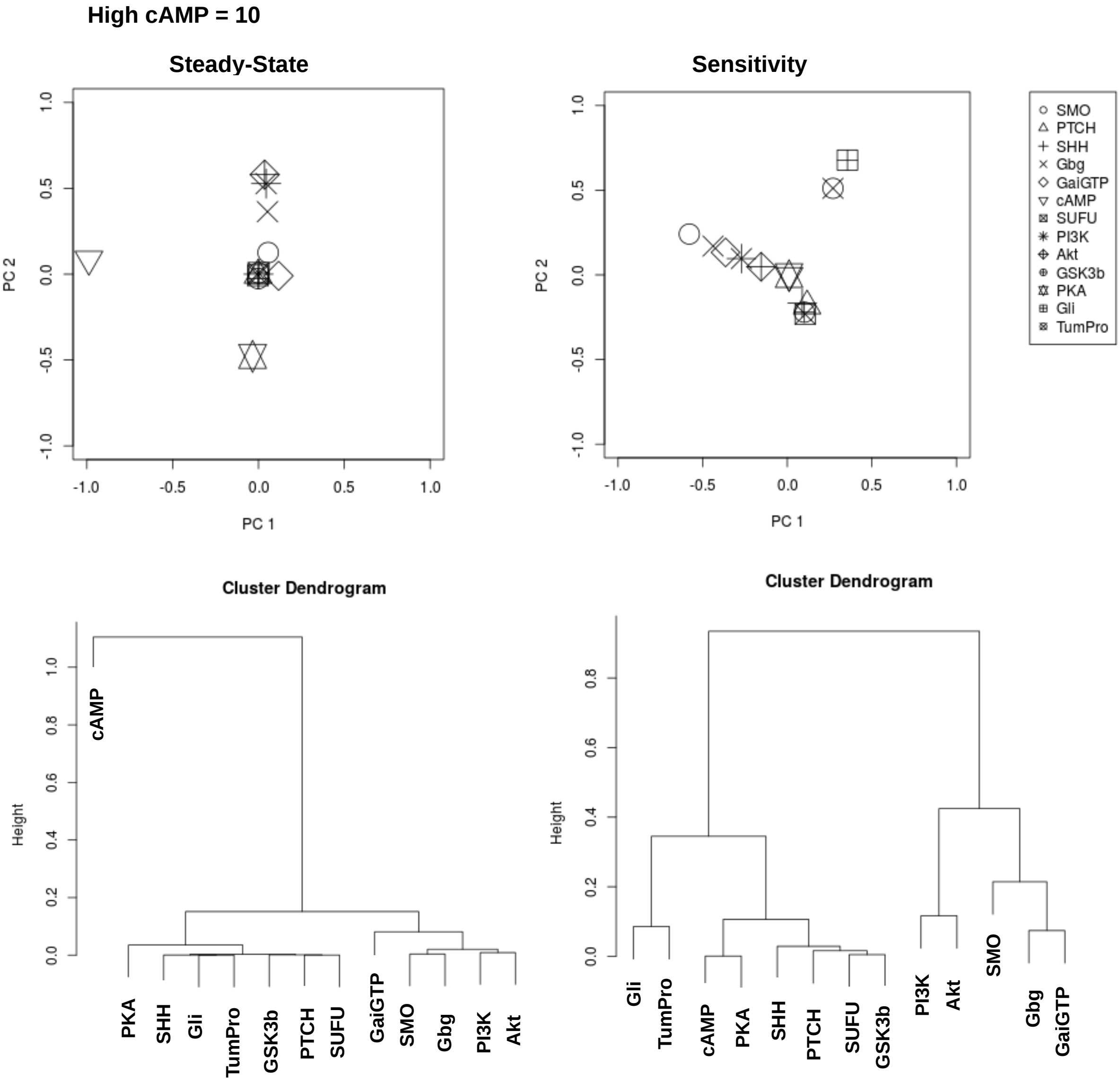

Supplementary Figure S3: **PCA at high levels of cAMP (strong PDEs inhibition).** Steady-state (left) and sensitivity (right) PCA of the network control analysis dataset presented in section “Signalling pathway analysis identified PI3K/Akt and PDEs as potential targets for treatment of BCC” at high cAMP. Hierarchical clustering dendrograms (bottom panels) on the PCA loadings (upper panels).

**Parameter Name**	**Signalling Pathway Analysis***	**Network Control Analysis***
$\beta(SMO)$	10^{-3} - $10^{-1}(100)$	$10^{-4} - 10^{+1}(1.2)$
$\beta(PTCH)$	10^{-3} - $10^{-1}(100)$	10^{-3}
$\beta(SHH)$	10^{-3} - $10^{-1}(100)$	10^{-3}
$\beta(Gbg)$	10^{-3} - $10^{-1}(100)$	10^{-3}
$\beta(GaiGTP)$	10^{-3} - $10^{-1}(100)$	10^{-3}
$\beta(cAMP)$	10^{-3} - $10^{-1}(100)$	10^{-3}
$\beta(SUFU)$	10^{-3} - $10^{-1}(100)$	10^{-3}
$\beta(PI3K)$	10^{-3} - $10^{-1}(100)$	$10^{-4} - 10^{+1}(1.2)$
$\beta(Akt)$	10^{-3} - $10^{-1}(100)$	10^{-3}
$\beta(GSK3b)$	10^{-3} - $10^{-1}(100)$	10^{-3}
$\beta(PKA)$	10^{-3} - $10^{-1}(100)$	10^{-3}
$\beta(Gli)$	10^{-3} - $10^{-1}(100)$	10^{-3}
$\beta(TumPro)$	10^{-3} - $10^{-3}(1)$	10^{-3}

Supplementary Table 1: **Model parametrization.** Activity units are arbitrary and in the context of signalling proteins roughly translate to the order of 1μM[1]. Values for the network end-points can only be appreciated by comparison.

References

[1] R. Milo, P. Jorgensen, U. Moran, G. Weber, and M. Springer. BioNumbers–the database of key numbers in molecular and cell biology. *Nucleic Acids Res.*, 38(Database issue):D750–753, Jan 2010. [PubMed Central:PMC2808940] [DOI:10.1093/nar/gkp889] [PubMed:19854939].
